# Supplementary material for: Modeling glioblastoma heterogeneity as a dynamic network of cell states
Source: Mol Syst Biol. 2021 Sep 16;17(9):e10105. doi: 10.15252/msb.202010105 (PMC8444284; doi:10.15252/msb.202010105)
Supplement: Supplementary file 6 — Source Data for Figure 5 [file MSB-17-e10105-s004.zip › Figure5A_sourcedata/GSEA_3017/nosigdb_stateA.GseaPreranked.1622795026485/RICHARDS_NOWAKOWSKI_SCIENCE_2017_MGE_RG1_UPREG.html]

Details for gene set RICHARDS\_NOWAKOWSKI\_SCIENCE\_2017\_MGE\_RG1\_UPREG[GSEA]

|  || Dataset | state53017 |
| Phenotype | NoPhenotypeAvailable |
| Upregulated in class | na\_neg |
| GeneSet | RICHARDS\_NOWAKOWSKI\_SCIENCE\_2017\_MGE\_RG1\_UPREG |
| Enrichment Score (ES) | -0.46144044 |
| Normalized Enrichment Score (NES) | -2.669101 |
| Nominal p-value | 0.0 |
| FDR q-value | 0.0 |
| FWER p-Value | 0.0 |
Table: GSEA Results Summary

  

Fig 1: Enrichment plot: RICHARDS\_NOWAKOWSKI\_SCIENCE\_2017\_MGE\_RG1\_UPREG      
 Profile of the Running ES Score & Positions of GeneSet Members on the Rank Ordered List

  

| PROBE | GENE SYMBOL | GENE\_TITLE | RANK IN GENE LIST | RANK METRIC SCORE | RUNNING ES | CORE ENRICHMENT || 1 | VIM |  |  | 46 | 0.543 | -0.0333 | No |
| 2 | FGFBP3 |  |  | 75 | 0.477 | -0.0491 | No |
| 3 | TNC |  |  | 159 | 0.402 | -0.1272 | No |
| 4 | RPS23 |  |  | 163 | 0.399 | -0.1181 | No |
| 5 | SOX6 |  |  | 234 | 0.358 | -0.1833 | No |
| 6 | WAC |  |  | 249 | 0.350 | -0.1878 | No |
| 7 | SLC25A5 |  |  | 266 | 0.340 | -0.1947 | No |
| 8 | BCAN |  |  | 276 | 0.334 | -0.1942 | No |
| 9 | NOC3L |  |  | 278 | 0.334 | -0.1850 | No |
| 10 | RPS6 |  |  | 306 | 0.324 | -0.2044 | No |
| 11 | MLLT10 |  |  | 311 | 0.322 | -0.1988 | No |
| 12 | HSPB1 |  |  | 312 | 0.322 | -0.1889 | No |
| 13 | SFRP2 |  |  | 320 | 0.319 | -0.1867 | No |
| 14 | PEG3 |  |  | 335 | 0.314 | -0.1922 | No |
| 15 | CREB3L2 |  |  | 348 | 0.310 | -0.1957 | No |
| 16 | BBIP1 |  |  | 401 | 0.293 | -0.2433 | No |
| 17 | CREB5 |  |  | 407 | 0.291 | -0.2398 | No |
| 18 | DOCK1 |  |  | 414 | 0.289 | -0.2374 | No |
| 19 | RPL41 |  |  | 436 | 0.285 | -0.2515 | No |
| 20 | BCHE |  |  | 456 | 0.280 | -0.2636 | No |
| 21 | SLITRK2 |  |  | 458 | 0.279 | -0.2561 | No |
| 22 | MNS1 |  |  | 464 | 0.278 | -0.2529 | No |
| 23 | AMMECR1 |  |  | 481 | 0.274 | -0.2619 | No |
| 24 | ZFP36L1 |  |  | 532 | 0.264 | -0.3082 | No |
| 25 | TCTN3 |  |  | 533 | 0.264 | -0.3001 | No |
| 26 | SOX9 |  |  | 577 | 0.254 | -0.3391 | No |
| 27 | NES |  |  | 603 | -0.252 | -0.3586 | No |
| 28 | CD99 |  |  | 604 | -0.252 | -0.3508 | No |
| 29 | ABAT |  |  | 617 | -0.259 | -0.3559 | No |
| 30 | TMTC2 |  |  | 633 | -0.263 | -0.3641 | No |
| 31 | ABCA1 |  |  | 647 | -0.268 | -0.3700 | No |
| 32 | PEA15 |  |  | 668 | -0.281 | -0.3831 | No |
| 33 | CKB |  |  | 676 | -0.287 | -0.3819 | No |
| 34 | HES1 |  |  | 736 | -0.331 | -0.4359 | No |
| 35 | PON2 |  |  | 743 | -0.338 | -0.4320 | No |
| 36 | MSI2 |  |  | 771 | -0.361 | -0.4503 | Yes |
| 37 | ITGB8 |  |  | 774 | -0.363 | -0.4413 | Yes |
| 38 | EPHA4 |  |  | 778 | -0.367 | -0.4332 | Yes |
| 39 | SPARC |  |  | 782 | -0.369 | -0.4251 | Yes |
| 40 | MLC1 |  |  | 783 | -0.369 | -0.4137 | Yes |
| 41 | NPC2 |  |  | 800 | -0.383 | -0.4193 | Yes |
| 42 | GPC1 |  |  | 801 | -0.384 | -0.4075 | Yes |
| 43 | CRIM1 |  |  | 805 | -0.388 | -0.3988 | Yes |
| 44 | MRC2 |  |  | 813 | -0.395 | -0.3942 | Yes |
| 45 | DDAH1 |  |  | 828 | -0.413 | -0.3967 | Yes |
| 46 | RORB |  |  | 830 | -0.413 | -0.3850 | Yes |
| 47 | PAM |  |  | 832 | -0.422 | -0.3731 | Yes |
| 48 | FILIP1L |  |  | 835 | -0.428 | -0.3621 | Yes |
| 49 | ZFP36L2 |  |  | 848 | -0.443 | -0.3615 | Yes |
| 50 | TFDP2 |  |  | 858 | -0.448 | -0.3575 | Yes |
| 51 | SLC35F1 |  |  | 871 | -0.482 | -0.3557 | Yes |
| 52 | ANXA2 |  |  | 872 | -0.482 | -0.3408 | Yes |
| 53 | PDPN |  |  | 890 | -0.529 | -0.3430 | Yes |
| 54 | GPC6 |  |  | 896 | -0.540 | -0.3318 | Yes |
| 55 | GPM6B |  |  | 897 | -0.542 | -0.3151 | Yes |
| 56 | ATP1B2 |  |  | 904 | -0.558 | -0.3044 | Yes |
| 57 | RAB31 |  |  | 916 | -0.586 | -0.2983 | Yes |
| 58 | GLUL |  |  | 920 | -0.612 | -0.2827 | Yes |
| 59 | TENM1 |  |  | 930 | -0.650 | -0.2724 | Yes |
| 60 | TTYH1 |  |  | 932 | -0.652 | -0.2534 | Yes |
| 61 | CLU |  |  | 938 | -0.685 | -0.2377 | Yes |
| 62 | CDH2 |  |  | 947 | -0.762 | -0.2229 | Yes |
| 63 | IFITM3 |  |  | 954 | -0.830 | -0.2038 | Yes |
| 64 | LYPD1 |  |  | 955 | -0.839 | -0.1780 | Yes |
| 65 | CNN3 |  |  | 961 | -0.883 | -0.1562 | Yes |
| 66 | ZBTB20 |  |  | 964 | -0.895 | -0.1307 | Yes |
| 67 | CDK6 |  |  | 966 | -0.920 | -0.1034 | Yes |
| 68 | SLC1A3 |  |  | 973 | -1.097 | -0.0761 | Yes |
| 69 | CYR61 |  |  | 978 | -1.296 | -0.0405 | Yes |
| 70 | IGFBP5 |  |  | 983 | -1.595 | 0.0044 | Yes |
Table: GSEA details [plain text format]

  

Fig 2: RICHARDS\_NOWAKOWSKI\_SCIENCE\_2017\_MGE\_RG1\_UPREG: Random ES distribution      
 Gene set null distribution of ES for **RICHARDS\_NOWAKOWSKI\_SCIENCE\_2017\_MGE\_RG1\_UPREG**
